# Obese patients with long COVID-19 display abnormal hyperventilatory response and impaired gas exchange at peak exercise

**DOI:** 10.2217/fca-2022-0017

**Published:** 2022-06-06

**Authors:** Mickael Rejaudry Lacavalerie, Sandrine Pierre-Francois, Moustapha Agossou, Jocelyn Inamo, André Cabie, José Luis Barnay, Rémi Neviere

**Affiliations:** ^1^Department of Neurosciences, Division of Functional Explorations, University Hospital of Martinique, Fort de France, 97200, France; ^2^Cardiovascular Research Team, Université des Antilles, Fort de France, EA, 7525, France; ^3^Department of Infectious Diseases, University Hospital of Martinique, Fort de France, 97200, France; ^4^Department of Pulmonary Diseases, University Hospital of Martinique, Fort de France, 97200, France; ^5^Department of Cardiology, University Hospital of Martinique, Fort de France, 97200, France; ^6^PCCEI, Université Montpellier, Université des Antilles, INSERM, EFS, Montpellier, 34394, France; ^7^Department of Physical & Rehabilitation Medicine, University Hospital of Martinique, Fort De France, 97200, Martinique

**Keywords:** aerobic capacity (VO_2_), chronic post-COVID-19 syndrome, hyperventilation, obesity, oxygen pulse, pulse oxymetry

## Abstract

**Aim:** To analyze the impact of obesity on cardiopulmonary response to exercise in people with chronic post-coronavirus disease 2019 (COVID-19) syndrome. **Patients & methods:** Consecutive subjects with chronic post-COVID syndrome 6 months after nonsevere acute infection were included. All patients received a complete clinical evaluation, lung function tests and cardiopulmonary exercise testing. A total of 51 consecutive patients diagnosed with chronic post-COVID-19 were enrolled in this study. **Results:** More than half of patients with chronic post-COVID-19 had a significant alteration in aerobic exercise capacity (VO_2_peak) 6 months after hospital discharge. Obese long-COVID-19 patients also displayed a marked reduction of oxygen pulse (O_2_pulse). **Conclusion:** Obese patients were more prone to have pathological pulmonary limitation and pulmonary gas exchange impairment to exercise compared with nonobese COVID-19 patients.

## Plain language summary

In this study, the cardiopulmonary response to exercise in people with chronic post-COVID-19 syndrome was analyzed. More than half of patients diagnosed with chronic post-COVID-19 had reduced exercise capacity 6 months after hospital discharge. In addition, patients with chronic post-COVID-19 syndrome who were overweight or obese displayed exaggerated hyperventilation along with an impairment of oxygenation at peak exercise.

Individuals with obesity have an increased risk of hospitalization, increased need for intensive care and increased risk of death [1–4]). As clinicians continue to learn about and treat coronavirus disease 2019 (COVID-19), growing evidence suggests that symptoms after acute infection may persist long after the acute infectious phase. Post-COVID-19 conditions are characterized by long-term consequences persisting or appearing after the typical convalescence period of COVID-19. The so-called chronic post-COVID-19 syndrome is defined as postacute COVID-19 syndrome with persistent symptoms after 12 weeks [5–7]. Reported symptoms span a large breadth of cardiopulmonary and neurologic complaints including fatigue, palpitations, chest pain and breathlessness [5–7]. As we are just beginning to understand the long-term implications of COVID-19 on health, old age, being female, poor prepandemic mental health, poor general health and being overweight or obese, confer an increased risk of developing chronic post-COVID-19 syndrome [6–8].

Respiratory symptoms dominate both the acute phase and longer-term sequelae of COVID-19 and exercise dyspnea and fatigue are the most common complaints [9]. Evaluation of aerobic capacity can provide further insights into the exercise intolerance commonly observed in patients with chronic post-COVID-19 [9–15]. Typical cardiopulmonary exercise testing (CPET) findings in chronic post-COVID-19 patients include a decrease in VO_2_peak, decreased anaerobic threshold, exaggerated hyperventilatory response, preserved ventilatory reserve and decreased oxygen extraction, all suggestive of skeletal muscle deconditioning [9–15]. Several CPET studies have been designed to conduct subgroup analyses of patients presenting with comorbidities such as obesity, cardiovascular conditions and chronic lung diseases. Whereas obesity is a well-recognized risk factor for severe COVID-19 and hospital admission for postacute COVID-19, whether obesity affects long COVID-19 cardiopulmonary response to exercise has not been extensively studied. The objective of this study was to determine cardiopulmonary function during exercise 6 months after hospital discharge for COVID-19 and to analyze the impact of obesity on cardiopulmonary response to exercise in these patients compared with non-COVID-19 obese people.

## Methods

### COVID-19 patients

Consecutive COVID-19 patients were included following evaluation at the University Hospital Martinique, France, from October 2020 to June 2021. Martinique is a French Caribbean island classified high in terms of global human development at the world level. Martinique has a population of 358,749 inhabitants (January 1, 2020). Patients were included during the third COVID-19 epidemic thread. A total of 11,739 COVID-19 cases were diagnosed in Martinique and 10% of patients were admitted to the University Hospital of Martinique. Patients with a reverse transcription-polymerase chain reaction (RT-PCR)-confirmed SARS-CoV-2 infection who were admitted to COVID-19 medical wards were included. Patients were included if they presented at the pluri-disciplinary consultation with persistent symptoms after a 6-month follow-up. Only patients with noncritical COVID-19 (i.e., mild disease, score: 3–4 on the WHO 9-point ordinal clinical progression scale) were included in this study [14]. Clinical examination and CT scans of the chest were normal at the time of the patient's inclusion. Criteria for chronic post-COVID-19 syndrome were a combination of symptoms including fatigue, shortness of breath, chest pain, palpitation and cognitive impairment [5]. Patients were invited to take part in an evaluation program that included lung function and cardiopulmonary exercise testing. No recommendations were given to the patients to refrain from taking their usual medications for diabetes or hypertension.

### Obese non-COVID-19 patients

During the same period of inclusion, functional parameters of obese non-COVID-19 patients with no history of cardiopulmonary diseases were included. Complete clinical and pulmonary and cardiovascular functional testing was performed for the purpose of preoperative evaluation before bariatric surgery. This group of patients represents a random sample of individuals who came for bariatric surgery at the same time chronic post-COVID-19 patients were recruited.

### Study design

Six months after hospital discharge, all patients received a complete clinical evaluation, spirometry and cardiopulmonary exercise testing. A statement on written informed consent in line with the Journal's policy on studies in human subjects was obtained from all patients. The study was conducted in accordance with the amended Declaration of Helsinki, registered in the Trials Registry and approved by the local Institutional Review Board.

### Spirometry & body plethysmography

Standard spirometry including forced vital capacity (FVC), forced expiratory volume in 1 s (FEV1) and total lung capacity (TLC) was measured by body plethysmography in a Jaeger-Masterlab cabin using guidelines of the American Thoracic Society (ATS) and the European Respiratory Society. Lung diffusion capacity for carbon monoxide (DLCO) was measured by the single-breath technique. A restrictive ventilatory pattern was defined as a combination of FEV1/FVC >0.70 and FVC <80% of predicted.

### Cardiopulmonary exercise testing

Cardiopulmonary exercise testing (Case, GE Healthcare, equipped with a PowerCube-Ergo, Ganshorn Medizin Electronic GmbH, Niederlauer, Germany) was performed according to standardized procedures on an electromagnetically braked cycle ergometer, as recommended by the ATS/American College of Chest Physicians (ACCP) [16]. Factors, such as age and BMI, were taken into account using the predicted VO_2_ Wasserman's equation for males: VO_2_max (L/min) = [0.79 × height − 60.7) × (50.72 − 0.372 × age) + 6 × (BMI − (0.79 × height − 60.7)] and Wasserman's equation for female: [(0.65 × height − 42.8) + 43) × (22.78 − 0.17 × age) + 6 × (BMI − (0.65 × height − 42.8)]. VO_2_peak was expressed as the percent of predicted. In addition, because VO_2_peak expressed relative to body weight (mL·kg^-1^ ·min^-1^) has been shown to underestimate VO_2_ in obese subjects, VO_2_peak was scaled relative to the body weight raised to the power of 0.75 (mL·kg^-0.75^·min^-1^) [16].

Following a 3-min warm-up, a progressively incremental (10 W/min) exercise was started until volitional fatigue. Subjects were continuously monitored using 12-lead ECG and pulse oximetry (SpO_2_). Breath-by-breath cardiopulmonary data were measured at rest, warm-up and incremental exercise testing. Minute ventilation (V_E_), oxygen uptake (VO_2_) and carbon dioxide output (VCO_2_) were recorded as concurrent 10-s moving averages and expressed as the percent of predicted values by Wasserman's equation. Calibration procedures for O_2_ and CO_2_ analyzers and the flow rate were run before each test as recommended by the manufacturer (Ganshorn Medizin Electronic GmbH, Niederlauer, Germany). In brief, calibration for O_2_ and CO_2_ analyzers was performed using two reference gases, pure air and a mixture of 16% O_2_ and 5% CO_2_. Flow rate calibration was performed using a calibrated 3L syringe. During the study period, mean values between qualified replicate tests performed weekly on control subjects were 2.5 ± 2.3%, 3.1 ± 1.2% and 2.5 ± 1.7% for peak VO_2_, VCO_2_ and V_E_, respectively. The ventilation anaerobic threshold was determined by the V-slope method [17,18]. Ventilatory efficiency, as indicated by the increment in V_E_ relative to VCO_2_ (V_E_-VCO_2_ slope) was calculated offline as a linear regression function using 10-s averaged values and excluding the nonlinear part of the relationship after the respiratory compensation point [17]. Peak oxygen pulse (O_2_ pulse) was calculated and expressed in ml/beat and as the percentage of the predicted value by dividing the predicted peak VO_2_ by the predicted peak heart rate. Assuming normal values of arterial oxygen content and C(a-v)O_2_ at peak effort, peak stroke volume in mm can then be estimated as (oxygen pulse/15) × 100, where oxygen pulse is in mm/beat [18]. Patient effort was considered maximal if two of the following occurred: predicted maximal work was achieved, age-predicted maximal heart rate was achieved, ventilatory O_2_ equivalent VE/VO_2_ >45 and respiratory exchange ratio (RER, i.e. volume of carbon dioxide produced/volume of oxygen consumed) >1.10, as recommended by the ATS/ACCP [17]. Symptoms and subjective ratings of perceived exertion were recorded to estimate exertion level.

### Statistical analysis

For all descriptive and inferential analyses, the assumption of normal distribution of the data was analyzed. Means and standard deviations are reported for normally distributed variables and median and min–max range are reported for nonnormally distributed variables. Categorical variables are presented as frequencies or percentages. Student *t-*tests and Chi-square tests were used for group comparisons. Based on early data on peak VO_2_ from COVID-19 patients, a sample size of at least 20 patients per group would be sufficient to detect a difference of 10% in predicted VO_2_ (a minimal clinically relevant difference in cardiopulmonary diseases) between groups (statistical power: 80%, alpha error: 5%). The level of statistical significance was set at p < 0.05. All statistical analyses were conducted using SAS software 9.4 for Windows (SAS Institute, NC, USA).

## Results

Using post-COVID-19 syndrome criteria, 60 consecutive patients with the diagnosis of chronic post-COVID-19 were enrolled. A total of 9 patients (15.0%) who were unable to perform exercise were excluded. The final population included 51 patients (Table 1). Dyspnea and fatigue were the most frequent symptoms of chronic post-COVID-19 syndrome. A restrictive ventilatory pattern was observed in 59% of cases. Detailed quantitative lung function parameters are displayed in Table 2. Altered aerobic capacity (VO_2_peak <75% of predicted values) was observed in 53% of cases. Mean aerobic capacity was significantly impaired (VO_2_peak = 16.2 ± 4.8 mL·kg^-0.75^·min^-1^, 72 ± 13% of predicted; Table 2). Characteristics of chronic post-COVID-19 patients were then stratified according to BMI below or above 30 kg/m^2^. Compared with nonobese COVID-19 patients (BMI <30 kg/m^2^), obese COVID-19 patients (BMI >30 kg/m^2^) had similar age, sex ratio, comorbidities and duration of hospital stay (Table 1). Compared with nonobese COVID-19 patients, obese COVID-19 patients had lower lung volumes (Table 2), while the lung transfer coefficient for carbon monoxide (KCO) was higher in obese patients. Despite similar VO_2_peak lowering, obese COVID-19 patients exhibited increased VE-VCO_2_ slope, reduced ventilatory reserve and lower SpO_2_ values compared with nonobese COVID-19 patients (Table 1). Amputated ventilatory reserve (<15%) and significant reduction (<3%) of SpO_2_ values were more frequent in obese patients compared with nonobese patients (39.4% vs 11.1%, p = 0.032; 51.5% vs 16.7%, p = 0.015, respectively).

**Table 1.** Pulmonary function and cardiopulmonary exercise testing in chronic post-COVID-19 patients.

Characteristics	All COVID-19 patients (n = 51)	Nonobese (n = 18)	Obese (n = 33)	p-value
Clinical variables				
Age, years (mean ± SD)	60 ± 11	64 ± 13	58 ± 10	0.071
Men, n (%)	30 (59)	12 (67)	18 (55)	0.295
BMI, kg/m^2^ (mean ± SD)	31 ± 6	25 ± 2	34 ± 5	**<0.001** ^†^
Presence of cardiovascular risk factors				
History of hypertension, n (%)	20 (39)	8 (44)	12 (36)	0.394
History of diabetes, n (%)	14 (28)	4 (22)	10 (30)	0.392
History of COPD, n (%)	5 (10)	3 (17)	2 (6)	0.230
Length of hospital stay for acute COVID-19				
Days (mean ± SD)	7.4 ± 2.1	7.4 ± 2.6	7.4 ± 1.8	0.955
Time from hospital discharge to functional evaluation				
Days (mean ± SD)	189 ± 12	187 ± 12	190 ± 13	0.389
Symptoms of chronic COVID-19				
Fatigue, n (%)	46 (90)	16 (89)	30 (91)	0.585
Dyspnea, n (%)	43 (84)	12 (67)	31 (94)	0.024^†^
Chest pain, n (%)	13 (25)	3 (17)	10 (20)	0.501
Pulmonary function test				
Predicted FEV_1_ (%)	79 ± 14	87 ± 13	75 ± 13	**0.002** ^†^
Predicted FVC (%)	77 ± 15	82 ± 16	74 ± 14	**0.045** ^†^
FEV_1_/FVC (%)	84 ± 8	84 ± 7	83 ± 9	0.641
FRC (%)	79 ± 19	81 ± 18	77 ± 19	0.514
TLC (%)	73 ± 12	79 ± 9	69 ± 12	**0.003** ^†^
ERV (%)	58 ± 27	67 ± 25	53 ± 28	0.080
RV (%)	80 ± 25	71 ± 25	86 ± 24	**0.043** ^†^
DLCO (%)	77 ± 13	77 ± 11	77 ± 14	0.882
KCO (%)	105 ± 12	100 ± 11	108 ± 12	**0.029** ^†^
Cardiopulmonary exercise testing				
Peak workload (% predicted)	54 ± 15	52 ± 14	56 ± 16	0.458
Peak VO_2_ (L.min^-1^)	1.44 ± 0.50	1.28 ± 0.41	1.53 ± 0.53	0.089
Peak VO_2_ (mL.kg^-0.75^.min^-1^)	16.2 ± 4.8	17.1 ± 4.5	15.7 ± 5.0	0.326
Peak VO_2_ (% predicted)	72 ± 13	70 ± 11	73 ± 14	0.436
Peak RER	1.12 ± 0.13	1.13 ± 0.12	1.12 ± 0.10	0.886
Peak VE/VO_2_	36 ± 6	35 ± 5	39 ± 7	**0.011** ^†^
Peak VE/VCO_2_	33 ± 6	32 ± 6	34 ± 6	0.261
Ventilatory reserve (%)	31 ± 20	40 ± 14	25 ± 21	**0.011** ^†^
VE VCO_2_ slope	32 ± 4	34 ± 6	31 ± 4	**0.045** ^†^
Peak O_2_ pulse (%)	66 ± 13	68 ± 12	66 ± 13	0.567
Peak heart rate (%)	87 ± 11	90 ± 11	85 ± 10	0.089
Peak heart rate used (%)	74 ± 22	80 ± 25	70 ± 21	0.135
Peak systolic pressure (mmHg)	197 ± 29	199 ± 27	197 ± 30	0.798
Peak diastolic pressure (mmHg)	98 ± 21	102 ± 23	97 ± 20	0.460
Peak SpO_2_ (%)	98 ± 21	98 ± 2	96 ± 3	**0.036** ^†^
Peak Borg scale dyspnea	5 [3–8]	5 [3–8]	5 [3–8]	0.538
Peak Borg scale leg fatigue	7 [3–8]	6.5 [3–8]	7 [3.5–8]	0.586

Data are presented as mean ± standard deviation (SD) and median (IRQ); peak heart rate (% predicted 220-age).

† Statistical significance set at p < 0.05.

AT: Anaerobic threshold; Borg: Modified Borg scale (0-10) for rate of perceived exertion scale; bpm: Beat per minute; COPD: Chronic obstructive pulmonary disease; DLCO: Diffusion capacity of the lung for carbon monoxide; ERV: Expiratory reserve volume; FEV_1_: Forced expiratory volume after 1 second; FRC: Functional residual capacity; FVC: Forced vital capacity; KCO: Transfer coefficient of the lung for carbon monoxide; RER: Respiratory exchange ratio; RV: Residual volume; SpO2: Pulse oximetry; TLC: Total lung capacity; VCO2: Pulmonary carbon dioxide output; VE: Minute ventilation; VO_2_: Oxygen uptake.

**Table 2.** Pulmonary function and cardiopulmonary exercise testing in obese chronic post-COVID-19 and obese non-COVID-19 patients.

	Obese COVID-19 patients (n = 33)	Obese non-COVID-19 patients (n = 29)	p-value
Clinical variables			
Age, years (mean ± SD)	58 ± 10	50 ± 13	0.008
Men, n (%)	18 (55)	6 (21)	0.009
BMI, kg/m (mean ± SD)	34 ± 5	41 ± 8	<0.001
History of hypertension, n (%)	12 (36)	12 (41)	0.796
History of diabetes, n (%)	10 (30)	12 (41)	0.431
Pulmonary function test			
Predicted FEV_1_ (%)	75 ± 13	74 ± 8	0.721
Predicted FVC (%)	74 ± 14	76 ± 8	0.501
FEV_1_/FVC (%)	83 ± 9	85 ± 4	0.274
FRC (%)	77 ± 19	76 ± 12	0.808
TLC (%)	69 ± 12	76 ± 9	**0.013** ^†^
Cardiopulmonary exercise testing			
Peak workload (% predicted)	56 ± 16	62 ± 12	0.104
Peak VO_2_ (L.min^-1^)	1.53 ± 0.53	1.68 ± 0.66	0.381
Peak VO_2_ (mL.kg^-0.75^.min^-1^)	15.7 ± 5.0	15.3 ± 2.7	0.702
Peak VO_2_ (% predicted)	73 ± 14	72 ± 14	0.780
Peak RER	1.12 ± 0.10	1.16 ± 0.10	0.831
Peak VE/VO_2_	39 ± 7	34 ± 5	**0.002** ^†^
Peak VE/VCO_2_	34 ± 6	31 ± 4	**0.026** ^†^
Ventilatory reserve (%)	25 ± 21	39 ± 16	**0.005** ^†^
VE VCO_2_ slope	31 ± 4	32 ± 6	0.438
Peak O_2_ pulse (%)	66 ± 13	76 ± 12	**0.003** ^†^
Peak heart rate (%)	85 ± 10	87 ± 7	0.3716
Heart rate reserve used (%)	70 ± 21	73 ± 15	0.525
Peak systolic pressure (mmHg)	197 ± 30	195 ± 32	0.080
Peak diastolic pressure (mmHg)	97 ± 20	93 ± 19	0.424
Peak SpO_2_ (%)	96 ± 3	98 ± 2	**0.004** ^†^
Peak Borg scale dyspnea	5 [3–8]	4.5 [3.5–8]	0.698
Peak Borg scale leg fatigue	7 [3.5–8]	6 [3–8]	0.766

Data are presented as mean ± standard deviation (SD); Student's *t*-tests were used for group comparisons.

† Statistical significance set at p < 0.05.

Borg: Modified Borg scale (0-10) for rate of perceived exertion scale; FEV1: Forced expiratory volume after 1 second; FRC: Functional residual capacity; FVC: Forced vital capacity; RER: Respiratory exchange ratio; SpO_2_: Pulse oximetry; TLC: Total lung capacity; VCO2: Pulmonary carbon dioxide output; VE: Minute ventilation; VO_2_: Oxygen uptake.

Descriptive characteristics for obese non-COVID patients are summarized in Table 2. Compared with obese non-COVID-19 patients, obese COVID-19 patients displayed exaggerated ventilatory drive, reduced ventilatory reserve, reduced O_2_ pulse and lower SpO_2_ values, with similar impairment of aerobic capacity (Table 2).

## Discussion

More than half of nonsevere hospitalized COVID-19 patients diagnosed with chronic post-COVID-19 displayed reduced aerobic capacity 6 months after hospital discharge in the current research. Despite similar VO_2_peak impairment compared with nonobese chronic post-COVID-19 patients, obese chronic post-COVID-19 patients displayed exaggerated hyperventilation as evidenced by increased ventilatory equivalent for oxygen (VE/VO_2_ ratio) and V_E_VCO_2_ slope along with abnormal pulmonary gas exchange at peak exercise. Functional lung tests of chronic post-COVID-19 patients were consistent with previous studies showing a reduction of lung volumes and impaired diffusion capacity for carbon monoxide (DLCO) along with lung transfer coefficient for carbon monoxide (KCO) above the upper limit of normal [19]. Recent insightful comments have suggested that the contrasting diffusion capacity and transfer coefficient changes may be the result of reduced alveolar volume, residual interstitial abnormalities and pulmonary vascular abnormalities [20].

These results are also consistent with previous studies showing that one-third of nonsevere COVID-19 survivors have a significant alteration in VO_2_peak after hospital discharge [15]. The present results highlight the deleterious impacts of obesity on ventilatory response and gas exchange at exercise in chronic post-COVID-19 patients. Indeed, the behavior of the pulmonary response at peak exercise in obese chronic post-COVID-19 patients was characterized by hyperventilation with amputated ventilatory reserve, along with significant fall in SpO_2_, which are all dysfunctional breathing responses to exercise and impairment of gas exchange. Exertional oxygen desaturation (SpO_2_) is an interesting finding that should be considered in rehabilitation programs for chronic post-COVID-19 in obese patients.

Despite similar aerobic capacity impairment, obese chronic post-COVID-19 patients had lower O_2_ pulse compared with obese non-COVID-19 patients, which suggests that extrapulmonary factors, especially cardiac dysfunction, may be considered in patients with chronic post-COVID-19. Hence, pulmonary dysfunction and gas transfer inefficiency may not be the sole reason for the abnormal exercise response in obese patients with chronic post-COVID-19, as reduced O_2_ pulse (a noninvasive surrogate of left ventricle stroke volume) indicated poor myocardial function. These findings should be integrated into the rehabilitation programs for these patients, particularly those who are overweight or obese.

### Study limitations

First, this observational study conducted at the University Hospital of Martinique is monocentric and retrospective. Second, cardiopulmonary exercise testing was performed on patients able to cycle and without contraindications such as unstable cardiovascular diseases, orthopedic impairment and mental impairment leading to inability to cooperate. Also, the high proportion of chronic post-COVID-19 patients displaying aerobic capacity impairment may be related to the preferential selection of CPET for patients with the more severe form of chronic post-COVID syndrome. Overall fitness status or physical activity of COVID-19 and non-COVID-19 patients were not evaluated as potential confounding factors for peak levels of VO_2_. Moreover, only patients with mild COVID-19 disease were studied and no further follow-up was performed. While displaying similar comorbidities, obese non-COVID-19 patients undergoing bariatric surgery were younger, mostly female and had higher BMI, making them a fairly weak comparison group for obese chronic post-COVID-19 patients.

## Conclusion

It has been previously shown that individuals with obesity may have an increased risk of hospitalization and death from acute COVID-19. Individuals with obesity may have a greater risk of hospital admission for chronic post-COVID-19 syndrome. This study was designed to better characterize the pulmonary, cardiac and functional capacity of SARS-CoV-2 survivors at 6 months by performing cardiopulmonary exercise testing. Six months after recovery from COVID-19, many patients are still affected by reduced maximal exercise capacity. Analysis of main CPET characteristics suggested that obese COVID-19 patients displayed exaggerated ventilatory drive, reduced ventilatory reserve and lower SpO_2_ values while having similar impairment of aerobic capacity. Thus, obese patients with long COVID-19 conditions should be considered at risk in exercise rehabilitation due to dysfunctional breathing response and impaired efficiency of pulmonary gas exchange.

**Summary points**

- Patients with chronic post-COVID-19 syndrome displayed abnormal cardiopulmonary response to exercise 6 months after hospital discharge that was characterized by impaired maximal aerobic capacity.
- Compared with normal-weight patients with chronic post-COVID-19 syndrome, patients who were overweight or obese displayed exaggerated hyperventilation as evidenced by increased ventilatory equivalent for oxygen (V_E_/VO_2_ ratio) and V_E_VCO_2_ slope, along with abnormal pulmonary gas exchange at peak.
- Compared with non-COVID-19 obese patients, chronic post-COVID-19 patients who were overweight or obese also demonstrated reduced oxygen pulse (a noninvasive surrogate of left ventricle stroke volume), which may suggest poor myocardial function.
